# Pain perception in people with Down syndrome: a synthesis of clinical and experimental research

**DOI:** 10.3389/fnbeh.2015.00194

**Published:** 2015-07-30

**Authors:** Brian E. McGuire, Ruth Defrin

**Affiliations:** ^1^School of Psychology and Centre for Pain Research, National University of IrelandGalway, Ireland; ^2^Department of Physical Therapy at the Sackler Faculty of Medicine and Sagol School of Neuroscience, Tel-Aviv UniversityTel-Aviv, Israel

**Keywords:** pain, Down syndrome, intellectual disability

## Abstract

People with an intellectual disability experience both acute and chronic pain with at least the same frequency as the general population. However, considerably less is known about the pain perception of people with Down syndrome. In this review paper, we evaluated the available clinical and experimental evidence. Some experimental studies of acute pain have indicated that pain threshold was higher than normal but only when using a reaction time method to measure pain sensitivity. However, when reaction time is not part of the calculation of the pain threshold, pain sensitivity in people with Down syndrome is in fact lower than normal (more sensitive to pain). Clinical studies of chronic pain have shown that people with an intellectual disability experience chronic pain and within that population, people with Down syndrome also experience chronic pain, but the precise prevalence of chronic pain in Down syndrome has yet to be established. Taken together, the literature suggests that people with Down syndrome experience pain, both acute and chronic, with at least the same frequency as the rest of the population. Furthermore, the evidence suggests that although acute pain expression appears to be delayed, once pain is registered, there appears to be a magnified pain response. We conclude by proposing an agenda for future research in this area.

## Pain in Intellectual Disability and Down Syndrome

Until recently, the commonly held view was that individuals with intellectual disability have decreased sensitivity to pain (e.g., Biersdorff, 1994; Feldt et al., 1998). This view was based on a number of factors such as the tendency of people with an intellectual disability not to report pain in potentially harmful situations and from observations of high rates of self-injurious behavior amongst some individuals with intellectual disability. However, recent reviews have cast doubt on these assumptions and have pointed instead to difficulties identifying pain in people with impaired (or different) means of communication and the presence of behavioral expressions of pain that may be variable and idiosyncratic (McGuire and Kennedy, 2013). Furthermore, the conceptual term “intellectual disability” captures a wide range of conditions, most of which are not well characterized and which are often of unknown etiology, making any generalization quite challenging. Notwithstanding these caveats, there are a number of reasons to suspect that people with intellectual disability may be at increased risk for experiencing chronic pain, including a possible heightened sensitivity to pain (Defrin et al., 2004), low levels of physical activity (Robertson et al., 2000), increased risk of accidental injury (Sherrard et al., 2001), reduced involvement in health decision making (McGuire et al., 2007), more physical co-morbidities (Baldridge and Andrasik, 2010) and reduced use of services for management of pain (McGuire et al., 2010).

While an intellectual disability is typically part of the clinical picture of Down syndrome, more is known about the specific features of Down syndrome than is the case when talking about intellectual disability as a more generic construct. People with Down syndrome may have specific risks for experiencing chronic pain because of increased risk of potentially painful conditions. These are synthesized here with approximate prevalence vales: congenital heart anomalies (15%), acquired cardiac disease (16%), chronic pulmonary changes (30%), osteoarthritic degeneration of the spine (32%), osteoporosis with resultant fractures of the long bones (55%) or vertebral bodies (30%), untreated atlanto-occipital instability (8%), eye problems (36%), celiac disease (11%), eczema (23%) (see for example; van Allen et al., 1999; Henderson et al., 2007; Hansdorfer et al., 2013).

For the purpose of this review we searched computerized databases (Pubmed, Medline, Scopus and Web of Science), published bibliographies of related topics, and references provided by colleagues. We limited our review to publications in the years 1960–2014. For this brief review, we have not attempted to conduct an exhaustive systematic review, but instead have selected literature germane to focus of our paper. Wherever possible in this paper, we will focus on the evidence regarding Down syndrome specifically, although in many studies the population described is a more general intellectual disability group of which some have Down syndrome. We will make the distinction wherever the literature allows us to.

## Pain Sensitivity in Down Syndrome

In order to determine whether pain sensitivity is altered in people with Down syndrome, it is necessary to have methods by which pain sensitivity can be measured accurately. Sensitivity to pain is measured experimentally by introducing subjects to stimuli of ascending or descending order until the boundary of pain is reached, a procedure termed “method of limits”. Another psychophysical method is the procedure of repeatedly introducing stimuli of various intensities and determining the threshold within the range of the stimulus that can almost never be detected, and that which is almost always detected as painful (“method of constant stimuli”). In both methods, pain threshold is defined as the smallest amount of stimulus energy (or intensity) necessary to evoke pain (Gescheider, 1985). The responses of the subjects to the stimuli in either method is based on self-report by way of verbal expression, body language, or withdrawal of the affected body part from the painful stimulus.

Only a few studies have actually measured pain threshold in individuals with Down syndrome. In the first study of its kind, Hennequin et al. (2000) evaluated pain threshold among 9 children and 17 adults with Down syndrome (age range 4–30 years) by measuring the time elapsed from the application of an ice cube on their wrist and temple to the first verbal expression of pain. The onset of verbal response was longer in individuals with Down syndrome compared with controls, suggesting a higher pain threshold (less sensitivity to pain) in the former. Valkenburg et al. (2015) has measured cold- and heat-pain thresholds, using computerized thermal stimulator among 21 children with Down syndrome (ages 10–15 years). The study measured the thresholds with the reaction-time dependent method of limits wherein subjects (or examiners) are required to press a switch the moment they perceive pain, thus ceasing the increase in stimulation intensity. Similar to Hennequin et al. (2000), the authors found higher thresholds compared to the participants’ siblings. However, in both of these studies, the pain threshold was affected by the reaction time of the individual and by the conduction velocity of the nervous system.

In an attempt to evaluate the effect of the pain measurement method on individual responses, Defrin et al. (2004) measured heat-pain threshold with two different methods; a reaction-time dependent method (method of limits) and a reaction-time free method (method of levels). In contrast to the method of limits, stimuli in the method of levels are predetermined and therefore their intensity is not affected by the subject’s performance nor by the conductance of the nervous system. Using a computerized thermal stimulator, 25 adults with an intellectual disability were tested, 14 of whom had Down syndrome (ages 22–56 years). When tested with the method of limits, individuals with Down syndrome exhibited pain threshold similar to that of age- and sex-matched cognitively intact controls. However, when tested with the method of levels, a significant group effect emerged wherein individuals with Down syndrome had a significantly lower pain threshold than controls (i.e., more sensitive to pain). These results suggested that individuals with Down syndrome are more sensitive to pain than normal but that slower reaction time gives the impression that pain threshold is higher than it actually is. Thus, this impression of reduced pain sensitivity appears to be an artifact associated with the method of measuring pain sensitivity and when reaction time is controlled for, there is evidence that people with Down syndrome are more sensitive to pain than average.

## Reaction-Time in Down Syndrome and its Relation to Pain Sensitivity

In evaluating the literature on pain sensitivity in Down syndrome, a question arises about the possible confound of: (a) capacity to report pain quickly; (b) possible altered somatosensory processes in Down syndrome. Some studies suggest that median nerve conduction velocities, (i.e., the speed at which action potentials evoked by electrical stimulation propagate along the median nerve) but not scalp-evoked potential latencies, were slower and their amplitude lower among children and adults with Down syndrome when compared with controls. This suggests impaired peripheral somatosensory function in Down syndrome (Kakigi, 1989; Brandt and Rosén, 1995).

In contrast, normal peripheral conduction velocity but prolonged latencies of somatosensory evoked potentials were recorded among infants or young adults with Down syndrome (Straumanis et al., 1973; Chen and Fang, 2005), suggesting that central conduction and/or processing of the nociceptive signals is delayed. The inter-hemispheric transmission time of adults with Down syndrome was also longer than in controls (Heath et al., 2007), further pointing to delays in central processing. Although these studies used innocuous stimuli, it is possible that conduction and processing of noxious stimuli may also be impaired and underlie the apparent decreased pain sensitivity when methods of sensitivity depend on conduction. Indeed, indirect evaluations of conduction velocity and reaction time based on responses to noxious thermal stimuli reveal slower times compared to controls in individuals with Down syndrome (Defrin et al., 2004; Valkenburg et al., 2011) and also compared to individuals with unspecified intellectual disability (Defrin et al., 2004). This may suggest a specific (slower) pain response in Down syndrome that is not attributable simply to the presence of intellectual disability. Consequently, measurements of pain that depend on reaction time or conduction velocity may portray a misleading hyposensitivity to pain due to delayed responses.

## Verbal Reports of Pain Among Individuals with Down Syndrome

Interviewing individuals with intellectual disability is important because, depending on their level of cognitive impairment, they can be the best source of information regarding their health. Despite this, people with intellectual disability are rarely involved in making important decisions regarding their health (McGuire et al., 2007). Little is known about the ability of individuals with Down syndrome to provide an adequate self-report of pain. This was evaluated in only a few studies. Zabalia and Corfec (2008) asked children and adolescents with Down syndrome to assess the pain of characters in pictures, using a FACES rating scale depicting pain responses and a visual-analog scale (a Likert-type scale). The children with Down syndrome were able to identify emotions and pain similar to children without Down syndrome, especially when using the FACES scale. de Knegt et al. (2013) introduced two rating scales; FACES and a numerical rating scale, to 106 adults with Down syndrome. The authors found that although participants better understood the FACES scale, 70% comprehended at least one of the two scales.

As part of a European initiative on pain in cognitive impairment (European Cooperation in the Field of Scientific and Technical Research [COST], 2015, TD-1005), our group has recently conducted an experimental study in which 29 adults with mild-moderate intellectual disability received pressure stimuli of various intensities during which time pain ratings were obtained using a pyramid pain rating scale. The pyramid scale is constructed of five pyramids of increasing sizes and heights expressing different pain intensities. The pain ratings of the nine participants with Down syndrome (age 31–36) correlated significantly with pressure intensity, suggesting that they could provide adequate ratings of their pain using this (graphical) scale (Benromano et al., 2015).

Results concerning pain self-report abilities of individuals with intellectual disability other than Down syndrome are inconsistent (e.g., Dagnan and Ruddick, 1995; McGrath et al., 1996; LaChapelle et al., 1999; Chibnall and Tait, 2001; Defrin et al., 2006). Generally, the reliability of self-report is usually inversely correlated with the intellectual disability level, with those less affected being more reliable in their ratings. Furthermore, graphical or 3 dimensional scales (e.g., the Poker Chip Tool) might be more suitable than two-dimensional verbal or numeric scales. Further study is needed to explore the best rating scale(s) for individuals with Down syndrome. In any case, the inability to report pain does not mean pain is not present (International Association for the Study of Pain, 1994) and the inability to use formal pain rating scales does not preclude the ability to provide free verbal report of the existence of pain, even as a gross indicator of the presence of pain.

## Behavioral Indicators of Pain

As not all individuals with intellectual disability can verbally communicate their pain, other indirect methods have been used to assess pain perception based on observation of manifestations that are considered indicators of pain. In very young children, intelligible verbal report of pain has not yet developed but basic vocalizations of pain may be present (such as crying) and there may be facial indicators or physiological indicators (such as heart rate or respiration rate).

Although frequently used in studies of general intellectual disability (LaChapelle et al., 1999; Nader et al., 2004; Dubois et al., 2010; Rattaz et al., 2013), only a few studies have examined behavioral or physiological indicators of pain specifically in Down syndrome. In an early study, Lind et al. (1970) found that babies with Down syndrome required more stimulation to provoke crying and had diminished visible responses to pain than control babies. More recently, Valkenburg et al. (2011) used the COMFORT-B scale (that includes manifestations such as alertness, respiratory response, body movements and crying) to assess post-operative pain among 76 new born babies and children with Down syndrome. Although mean COMFORT-B scores of children with Down syndrome were higher than comparable controls, the scores did not differ significantly between the groups. Aguilar Cordero et al. (2015) found that behavioral (e.g., crying) and physiological responses (oxygen saturation, heart rate, blood pressure) following vein/heel puncture were slower among 20 new born babies with Down syndrome and not as clearly defined as that of babies without Down syndrome. However, when pain was finally perceived, it persisted for a longer time among infants with Down syndrome. As with sensory testing, it appears that physiological manifestations of pain in new born babies with Down syndrome emerge more slowly than normal, but once registered, they may represent enhanced or prolonged pain experience. This may suggest either a magnified nociceptive process or a delayed or inefficient inhibitory response.

Facial expressions of pain following pressure stimuli among individuals with intellectual disability have recently been analyzed by our group in the aforementioned experimental study (Benromano et al., 2015), using the Facial Action Coding System (FACS; Ekman and Friesen, 1978). The nine participants with Down syndrome had significantly increased facial expressions compared to cognitively intact controls, both at baseline and throughout stimulation intensities. FACS scores correlated with stimulation intensity, suggesting that facial expressions can reliably indicate the intensity of pain among individuals with Down syndrome (Benromano et al., 2015). However, as there are also increased facial expressions at rest that could be interpreted as pain expression, additional measures of pain are advised. Kyrkou (2005) noted that a pale face and restlessness indicated the presence of menstrual pain among women with Down syndrome. We could not find additional studies that focused on facial/bodily expressions of pain specifically in Down syndrome. Nevertheless, several studies included people with Down syndrome among the tested populations and reported that as a whole, individuals with intellectual disability exhibit significant elevations in facial expressions/bodily movements during painful events as compared to baseline (e.g., Breau et al., 2002; Benini et al., 2004; Defrin et al., 2006; Dubois et al., 2010).

Some authors noted that individuals who were unable to verbalize their pain tended to exhibit atypical pain expressions such as freezing, smiling and hand flapping/rubbing (Defrin et al., 2006; Dubois et al., 2010). Such expressions, which are unexpected in the context of pain, may mislead observers to think that the individual is not in pain. Thus, measurements of behavioral indicators of pain might prove useful in quantifying pain among individuals with Down syndrome, but the optimal scale for doing so has yet to be determined.

## Caregiver Evaluation of Pain

A number of studies have attempted to estimate the extent of pain in people with general intellectual disability based on caregiver report. For example, two recent studies estimated that 13–15% of people with intellectual disability have chronic pain based on caregiver report (McGuire et al., 2010; Walsh et al., 2011), but concluded that pain was likely to be under-recognized and under-treated as a result (McGuire et al., 2010). This conclusion was based on the fact that third party evaluation of pain is very challenging, even for parents. For example, around 30% of parents of children with Down syndrome had difficulty perceiving if their child was in pain and 70% of the parents had difficulty identifying the location of the pain (Hennequin et al., 2003). The likelihood of parents reporting difficulty in discerning if and where their child with Down syndrome had pain was *greater* than for a sibling without Down syndrome. It is noteworthy, however, that parents of children with intact cognition may also experience difficulties in identifying the amount of pain experienced by their children (e.g., Jylli and Olsson, 1995; Chambers et al., 1998; Larochette et al., 2006). Thus, while parents are more familiar with their child’s typical pain reactions than other care takers, parents may still underestimate and overestimate pain, even in verbal children.

Davies (2010) recently reported that parents of children with Down syndrome assessed their child’s pain through the child’s verbalizations (words, showing pain location and crying), behavioral expressions (changes in usual activities, seeking closeness to the parent) and emotional changes (e.g., anger, fear, frustration and acting out). The parents reported that they also assessed pain based on their beliefs that the child was less verbal, slower to complain, and less bothered by pain than siblings. In another study, parents reported in 66% of the cases that their child was less sensitive to pain than normal, although there is some evidence that children with Down syndrome are more sensitive to heat/cold pain (Valkenburg, 2012). While knowledge of the idiosyncratic behaviors of their children will facilitate parents in recognising pain in their child with Down syndrome, this is more challenging for other caregivers such as teachers or health professionals. The unique pain expression of some people with Down syndrome may mislead caregivers.

## Imaging Studies in Down Syndrome and Implications for Pain Perception

Magnetic resonance imaging (MRI) studies reveal distinctive alterations in brain anatomy among individuals with Down syndrome. For example, there is evidence of smaller overall brain volume, disproportionately smaller cerebellar volume and relatively larger subcortical gray matter volume in people with Down syndrome compared to controls (Pinter et al., 2001). Aging occurs prematurely in Down syndrome and manifests in neuropathological atrophies typical of Alzheimer’s disease including, but not restricted to, reduction in hippocampal, parietal, orbitofrontal, lingual and post central volume (Teipel et al., 2004; Teipel and Hampel, 2006; Koran et al., 2014). The effects of these neuropathological changes on pain perception and behavioral expression of pain in Down syndrome is not known.

Functional connectivity MRI studies reveal higher regional connectivity in the ventral brain system (the amygdala and anterior temporal region and the ventral aspect of the anterior cingulate and frontal cortices) and lower connectivity in dorsal executive networks (dorsal prefrontal, anterior cingulate and posterior insula cortices; Pujol et al., 2015). These changes may affect the experience of pain. For example, the orbitofrontal cortex is involved in pain modulation via brain stem structures (Lorenz et al., 2003; Zeidan et al., 2011); reduced volume and connectivity of which may reduce pain modulation and thus increase the intensity of perceived pain among individuals with Down syndrome. Furthermore, structural, and related functional alterations in the insular and cingular as well as somatosensory cortices may induce alterations in processing of the sensory and affective aspects of pain (Davis and Moayedi, 2013). The connectivity increases and decreases found in Down syndrome are thought to account for reduced adaptive behavior, which in turn is related to communication skills (Pujol et al., 2015) and may thus also account for delayed and altered behavioral responses to pain, as described above.

## Modifying Pain Experience in People with Down Syndrome

Current models of pain conceptualize pain perception as being the consequence of integrating several sources of information including sensory information, cognitive appraisals of the pain, emotional responses, behavioral responses and social context. Thus far, both experimental and clinical studies of pain perception in people with Down syndrome have tended to focus on the sensory component of pain. Yet, in the broader population, many studies have evaluated methods for assisting with modifying both acute and chronic pain experience. For example, attention diversion is a well-established method of coping with pain (e.g., Van Damme et al., 2010) as is cognitive behavioral therapy (CBT; e.g., Eccleston et al., 2012) whereby cognitions and behaviors are modified in order to enhance pain coping. However, virtually no studies have evaluated these methods in people with Down syndrome or intellectual disability more generally, despite the fact that modified CBT has been shown to be effective for treating people with intellectual disability with depression, anxiety and anger problems (McGuire and Kennedy, 2013).

A few notable exceptions have looked at these methods for managing pain in people with an intellectual disability (although not Down syndrome *per se*). For example, a case report study of a person with a mild intellectual disability who had chronic pain indicated that psychological treatments may be of benefit (Lewis et al., 2007). A significant development has been the production of a CBT-based treatment manual (“*Feeling Better*”) designed to be used by caregivers to assist people with intellectual disability in developing pain self-management strategies (McManus and McGuire, 2010). In a case series, the authors of the treatment manual reported some preliminary evidence of the effectiveness of the programme (McManus and McGuire, 2014) but noted that more research is needed, including controlled clinical trials. Subsequently, a trial protocol has been registered (Kennedy et al., 2014) to evaluate the effectiveness of the *Feeling Better* programme for management of menstrual pain in young women with an intellectual disability. Treatment outcomes have not yet been reported but will be important as the first controlled trial to evaluate psychological management of pain for people with an intellectual disability.

## Animal Models

Studies on animal models of Down syndrome (e.g., the Ts65Dn and APP-SOD1 mice) also indicate delayed response to noxious stimuli compared to control mice (Martínez-Cué et al., 1999; Kotulska et al., 2011). At the same time, there is evidence of increased tissue pathology after induced damage in transgenic animals compared to controls, evident by more prominent neuroma formation, decreased motor neuron survival and impaired regeneration capacity (Kotulska et al., 2011). These studies imply that while Down syndrome is associated with slower conduction of noxious stimuli which may affect the animal’s pain behavior, the development of pathology following tissue damage is not delayed and may even be enhanced.

In another study, the response of Ts65Dn mice to neurotrophic factors such as nerve growth factor (NGF) was abnormal (Seo and Isacson, 2005). Although the consequence of this finding is not clear yet, neurotrophic factors were found to promote neuroma formation and enhance pain sensation, potentially underlying the changes found in individual pain thresholds. Further animal studies are needed to determine whether and which alterations exist in the conduction of noxious stimuli in Down syndrome.

### Conclusions and Recommendations for Future Studies

On the basis of the limited data on sensory and behavioral testing, we tentatively conclude that individuals with Down syndrome are more sensitive to pain than normal. The evidence suggests that although pain expression appears to be delayed, once pain is registered, there appears to be a magnified pain response. This conclusion corresponds with imaging studies showing differences in structures involved in pain modulation (e.g., frontal cortex) as well as structures involved in pain processing (e.g., cingulate, insula and sensory cortex). Still, some inconsistency exists in pain threshold measurements that may reflect interruption in peripheral conduction and central processing of sensory signals, especially if pain threshold is measured with methods that include reaction time. While such alterations have been reported for innocuous stimuli, studies are needed to prove that such alterations indeed occur in nociceptive pathways. Due to the possibility of delayed reaction time, measuring pain threshold with methods that bypass this limitation, i.e., reaction-time free methods, is preferable. However, pain threshold measurement is suitable only for individuals with mild and perhaps moderate cognitive impairment. Thus, the use of indirect indices of pain is necessary. Additional studies are needed in order to explore which indices best reflect pain in Down syndrome.

While individuals with Down syndrome are at increased risk to experience pain due to congenital and acquired abnormalities and environmental risk factors (e.g., higher risk of accidental injury), they typically have difficulty in expressing their pain and their caregivers face great challenges in identifying and quantifying pain. Thus, from a clinical point of view, it is imperative to investigate pain processing and pain expression of individuals with Down syndrome in both the experimental and clinical setting. Until optimal tools are available for this purpose, caregivers should take into consideration unexpected and sometimes seemingly ambiguous responses to painful incidents. We have previously advocated the use of more than one source of information to identify pain, in order to increase the reliability of the information obtained (McGuire and Kennedy, 2013).

Finally, people with Down syndrome exhibit evidence of premature aging and a greatly increased risk of developing Alzheimer disease (Zigman and Lott, 2007). In the general population, chronic pain is known to affect some 30–50% of people with Alzheimer disease (e.g., Shega et al., 2004; Zwakhalen et al., 2009), and within that population there are enormous challenges in identifying the presence of pain (Corbett et al., 2014) so as to implement an appropriate plan for pain management. No studies have yet examined the problem of pain in people with Down syndrome who also have evidence of onset of Alzheimer-related dementia. This “double jeopardy” represents a major challenge for both researchers and clinicians, but is an important area for future research.

In concluding, we propose the following agenda for future research in the area:

In the clinical domain:

1. More epidemiological studies on the prevalence and profile of chronic pain in people with Down syndrome.
2. Further evaluation of observer-based and self-report pain assessment tools.
3. Better understanding of potentially different pain expression based on the type of pain (e.g., neuropathic, inflammatory) or derivation of pain (e.g., post surgical (for example, tonsils, hip replacement), gastrointestinal, dental etc.).
4. The perception of pain associated with self-injury in low functioning persons with Down syndrome.
5. Evaluation of how the presence of dementia affects the manifestation of pain in people with Down syndrome.
6. A greater emphasis on evaluating pain management interventions, including self-management (psychological coping strategies).

In the experimental domain:

1. Further evaluation of observer-based and self-report pain assessment tools using calibrated noxious stimuli of varying intensities.
2. Measuring physiological and electrophysiological reactions to experimental pain that may potentially replace self report, including but not restricted to heart-rate variability and electromyography (EMG) and electroencephalogram (EEG).
3. Measuring conduction velocity and reaction time to noxious stimuli.
4. Studying pain perception using event related potentials (ERP) and functional magnetic resonance imaging (fMRI) that enable the association between noxious stimulation and activation in specific brain regions of interest.

## Conflict of Interest Statement

The authors declare that the research was conducted in the absence of any commercial or financial relationships that could be construed as a potential conflict of interest.
